# Pharmacokinetics of Depemokimab Delivered by Safety Syringe Device or Autoinjector in Healthy Adults: A Phase 1, Single‐Dose Study

**DOI:** 10.1002/cpdd.1506

**Published:** 2025-01-28

**Authors:** Stein Schalkwijk, Chiara Zecchin, Anusmita Sen, Sei Choi, Kai Wang, Jeff Min, Brian Spears

**Affiliations:** ^1^ Clinical Pharmacology Modelling and Simulation GSK London UK; ^2^ Clinical Pharmacology Modelling and Simulation GSK Stevenage Hertfordshire UK; ^3^ Biostatistics GSK Bengaluru India; ^4^ Global Clinical Development GSK Ottawa Canada; ^5^ Biomarker and Bioanalytical Platform GSK Collegeville PA USA; ^6^ Clinical Research Respiratory GSK Waltham MA USA; ^7^ Clinical Research Unit PPD Austin TX USA

**Keywords:** biologics, depemokimab, drug metabolism, pharmacokinetics, self‐administration

## Abstract

This Phase I, randomized, multicenter, open‐label, parallel‐group, single‐dose study assessed the relative bioavailability of the anti–interleukin‐5 antibody depemokimab (100 mg) when administered subcutaneously via either a safety syringe device (SSD) or an autoinjector (AI). Healthy adult participants were randomized I:I to SSD or AI treatment arms and I:I:I to the injection site (upper arm, abdomen, or thigh). Participants were followed up for 30 weeks; blood samples were collected for pharmacokinetic (PK) assessment before dosing on Day 1 and up to Week 26. Depemokimab concentration profile as measured by plasma maximum concentration (C_max_), the area under the concentration–time curve from time zero extrapolated to infinity (AUC_0‐inf_), PK parameters, immunogenicity, and safety were assessed. Overall, 140 participants were enrolled (n = 70 per arm). Mean plasma concentration‐time profiles of depemokimab were similar in both treatment arms, regardless of the injection site, adjusted geometric mean AI:SSD ratios for C_max_ and AUC_0‐inf_ were 1.03 and 1.03, respectively, with all 90% confidence intervals within the bioequivalence bounds of 0.80‐1.25. PK parameters were comparable across treatment arms. Treatment‐related adverse events were reported in 19% of SSD and 20% of AI participants, with headache being the most common across both arms; no adverse events led to study withdrawal. These results support the use of either SSD or AI for subcutaneous administration of depemokimab.

Anti–interleukin (IL)‐5/IL‐5 receptor therapies are now a cornerstone of asthma management for appropriate patients who continue to exacerbate despite optimized standard of care, with treatment options including reslizumab, mepolizumab, and benralizumab.^1^ Reslizumab and mepolizumab function by binding the circulating IL‐5 ligand to inhibit signaling, while benralizumab binds the IL‐5 receptor on basophils and eosinophils, resulting in their depletion via antibody‐dependent cell‐mediated cytotoxicity.^1^ Reslizumab is administered intravenously every 4 weeks, while mepolizumab and benralizumab require subcutaneous (SC) administration every 4 weeks and 4‐8 weeks, respectively.^1^ In survey and discrete choice experiment studies across multiple therapeutic areas, including severe asthma, patients and physicians reported strong preference for less frequent dosing schedules.^2^ Depemokimab is an ultra‐long‐acting biologic targeting human IL‐5, showing approximately 29‐fold increased potency and about a 2‐fold reduction in clearance compared with mepolizumab, as demonstrated in a single‐dose pharmacokinetic (PK) and pharmacodynamics studies in cynomolgus monkeys (GSK data on file). The safety, tolerability, and PK of SC depemokimab (2‐300 mg) have previously been assessed in a first‐in‐human study including patients with mild to moderate asthma and a blood eosinophil count of 200 cells/µL or greater at screening. Depemokimab was shown to be well tolerated, with linear and dose‐proportional PK properties, with a longer half‐life across all doses (38‐53 days) compared with the half‐lives of mepolizumab and benralizumab (16‐22 and 15.5 days, respectively), enabling a 6‐month dosing schedule with depemokimab.^3^, ^4^

The first‐in‐human study of depemokimab was conducted using SC administration syringes prepared from vials by laboratory and pharmacy staff.^3^ Most approved biologics for severe asthma can be administered subcutaneously (either at home or by a health care professional) through a prefilled disposable syringe, which can be assembled in a safety syringe device (SSD) or an autoinjector (AI).^1^ With an SSD, the needle is inserted into the skin and the medication is injected by depressing the plunger, following which the exposed needle automatically retracts into the body of the syringe.^5^, ^6^ By comparison, the needle in an AI is not visible to patients, and self‐injection is automated.^6^ Both devices allow convenient drug administration. However, individual patient or health care professional preferences may vary based on factors such as ease of use and injection site pain.^7^, ^8^, ^9^ To enable patients and health care professionals to choose their preferred device while ensuring equivalent medication exposure, relative bioavailability with the different approaches must be assessed in comparative studies.^10^, ^11^ This Phase I study sought to compare the relative bioavailability of depemokimab 100 mg SC following a single dose administered via SSD or AI in healthy participants.

## Methods

### Study Design and Procedures

This was a Phase I, randomized, multicenter, open‐label, parallel‐group, single‐dose study in healthy adult participants (GSK 214099; NCT05602025), conducted across 3 PPD clinics in the United States (Orlando, FL; Las Vegas, NV; Austin, TX). Participants were randomized (1:1) to receive single‐dose depemokimab 100 mg SC via SSD or AI, stratified according to body weight (less than 70 kg, 70 or greater to less than 80 kg, and 80 kg or greater) to minimize the potential for confounding factors. The injection site was randomized (1:1:1) to the upper arm, abdomen, or thigh. Eligible participants were admitted to a clinical research unit for dosing and monitored for 48 h; after discharge, they were followed via regularly scheduled outpatient visits, with a final telephone follow‐up at 30 weeks.

This study was conducted in accordance with the protocol and consensus ethical principles derived from international guidelines, including the Declaration of Helsinki, alongside other applicable laws and regulations. All participants signed a statement of informed consent before enrollment. All relevant study documents for each site were reviewed and approved by the central institutional review board of Advarra Inc., Columbia, Maryland, before study initiation.

### Participants

Key eligibility criteria for inclusion were healthy adults (18‐50 years of age) based on medical evaluation, body weight of 50 kg or greater, and body mass index (BMI) between 19 and 30 kg/m^2^ (inclusive). Women who had the potential to become pregnant were required to use a form of highly effective contraception.

The main exclusion criterion was history/presence of current cardiovascular, respiratory, hepatic, renal, gastrointestinal, endocrine, hematologic, or neurologic disorders capable of significantly altering the PK of drugs, constituting a risk when taking the study intervention, or interfering with the interpretation of data. Other exclusion criteria included allergy/intolerance to a monoclonal antibody or biologic or history of clinically significant multiple or severe drug allergies/intolerance; current evidence or recent history of parasitic infection or vasculitis; a positive prestudy drug/alcohol screen or a history (or suspected history) of alcohol misuse or substance abuse; clinically significant abnormalities identified during screening (with a focus on blood pressure, alanine aminotransferase [ALT], bilirubin, and QT interval corrected for heart rate using Fridericia's formula [QTcF]); positive test for severe acute respiratory syndrome coronavirus‐2 at screening; and recent prior or concurrent clinical study experience.

### Endpoints

To evaluate the relative bioavailability of depemokimab delivered via SSD or AI, the endpoints were the assessment of depemokimab maximum observed concentration (C_max_) and area under the concentration–time curve (AUC) from time zero extrapolated to infinity (AUC_0‐inf_) in plasma following single‐dose administration. Additionally, the PK parameters plasma AUC from time zero to time of the last quantifiable concentration (AUC_0‐t_), time at which C_max_ was observed (T_max_), apparent clearance following subcutaneous dosing (CL/F), apparent volume of distribution following subcutaneous dosing (Vz/F), terminal phase half‐life (T_1/2_), percentage of AUC_0‐inf_ obtained by extrapolation (%AUC_ex_), immunogenicity (ie, presence of antidrug antibodies [ADAs] and neutralizing antibodies [NAbs] to depemokimab) and safety (ie, adverse events [AEs], severe AEs [SAEs], change from baseline to Week 26 in laboratory parameters, hepatobiliary laboratory abnormalities, and electrocardiogram values) were also assessed. Specific AEs of special interest (AESIs) included Type I/III hypersensitivity reactions (allergic/immune complex disease or vasculitis, respectively) and local injection site reactions. Other endpoints included blood eosinophil counts and ratio to baseline over time, depemokimab measured concentration by injection site, depemokimab plasma C_max_ and AUC_0‐inf_ by injection site, and device functionality.

### Bioanalytical Method

Depemokimab plasma concentration was determined in plasma samples using a fully validated bioanalytical method by Frontage Laboratories, Inc. (Exton, PA) under the control of Bioanalysis, Immunogenicity and Biomarker, GSK. PK blood samples were collected before dosing on Day 1, and at 2 and 8 hours after dosing, then at Days 2, 3, 5, 8, 15, 29, 57, 85, 127, 169, and 183. Depemokimab concentrations in human K2EDTA acid plasma were quantified by a sandwich‐based ligand‐binding electrochemiluminescence (ECL) assay. Meso Scale Discovery (MSD) streptavidin‐coated plates were coated with biotinylated human IL‐5 protein in the presence of a blocking buffer, following which standards, controls, and samples were applied. After incubation with the detection antibody ruthenylated mouse anti‐human immunoglobulin G, plates were read in the MSD reader. The assay detection range was 100‐5000 ng/mL (lower limit of quantification to upper limit of quantification). The measured within‐run and between‐run precision of the assay was 8.2% or less and 10.4% or less, respectively. The measured overall accuracy was −3.6% to −8.6%.

### Immunogenicity Assessment

Immunogenicity blood samples were collected before dosing on Day 1, then on Days 29, 57, 85, and 183. For the determination of anti‐depemokimab antibodies in human serum, an initial acid dissociation for target removal with the biotinylated drug was followed with a homogeneous bridging assay format using biotinylated and ruthenylated drug conjugates, along with a target‐blocking antibody. The resulting immune complex bridge was detected with ECL using the MSD platform. The assay was developed and validated at GSK and then transferred and validated at Frontage Laboratories, Inc. (Exton, PA). To determine ADA positivity, a tiered analyses approach was used for immunogenicity assessment, including a validated binding ADA assay (screening, confirmation, and titration assays) and a validated NAb assay. For the ADA assay, samples were classed as presumed positive when their ECL value exceeded or was equal to the plate‐specific screening cut point (SCP; calculated on the basis of the SCP factor [value 1.07] multiplied by mean ECL value of the accepted negative controls on each plate). Positive status was confirmed in the confirmation runs when percentage inhibitions were 18.41% or greater (confirmatory cut point) and mean ECL value of depemokimab unspiked preparations were equal to or greater than the plate‐specific SCP. With a rabbit polyclonal‐positive control, the mean interpolated screening and confirmation sensitivities were 8.7 and 13.1 ng/mL, respectively. For drug tolerance, 100 ng/mL of positive control screened positive with 20 µg/mL of drug. The intra‐assay and interassay screening precision were 3.7% or less and 14.4% or less, respectively.

### Sample Size and Statistical Analyses

Approximately 140 participants were planned to be enrolled (accounting for a dropout rate of 10% until Week 18) to provide at least 126 evaluable participants (63 for each treatment arm). Assuming a within‐subject correlation of 0.8 between C_max_ and AUC_0‐inf_, and a true ratio of 1, a sample size of 63 per treatment arm would provide 90% power to observe the geometric confidence interval (CI) of the geometric mean ratio (GMR) within the bioequivalence range (0.80‐1.25) for both parameters. Arithmetic mean data are also presented. All study population and safety analyses were conducted in the safety population (ie, all participants who received 1 or more doses of the study intervention). PK analyses were conducted in the PK population (ie, participants in the safety population, for whom 1 or more evaluable PK samples were obtained and analyzed).

PK parameters were derived from the actual sampling times using noncompartmental methods with Phoenix WinNonlin version 8.0 or higher (Certara). The analysis of C_max_ and AUC_0‐inf_ was performed on the natural logarithms of the above parameters using fixed‐effects analysis of the covariance model, with treatment and injection site as categorical covariates and baseline weight as a continuous covariate fitted on the log scale to incorporate allometry. The estimates of the geometric means were therefore adjusted for injection site (abdomen, arm, thigh) and baseline weight (log scale), controlling for any imbalances between treatment groups. Point estimates and 90% CIs for treatment differences on the log scale derived from the model were exponentiated to obtain estimates for GMRs and CIs on the original scale.

Summary statistics were provided for PK endpoints AUC from time zero to time of the last quantifiable concentration, time at which C_max_ was observed, apparent clearance following subcutaneous dosing, apparent volume of distribution following subcutaneous dosin, terminal phase half‐life and %AUC_ex_ for each treatment arm. Immunogenicity was assessed by a tiered analysis approach (an ADA binding assay that includes a screening assay, a confirmation assay, a titration assay; and an NAb assay). Safety, blood eosinophil counts, and ratio to baseline over time; depemokimab measured concentration by injection site; and depemokimab plasma C_max,_ and AUC_0‐inf_ by injection site and device functionality were summarized using descriptive statistics.

## Results

### Participant Population

Of the 263 participants screened, 140 were enrolled (n = 70 in each arm). Most participants completed the study, 1 participant (1%) in the SSD and 1 (1%) in the AI arm were withdrawn due to loss of follow‐up and participant relocation, respectively. Overall, 75 participants (54%) were female and the median (minimum–maximum) age at screening was 36.0 years (18.0‐50.0; Table S1). The majority of participants (63%) were White. Demographic characteristics were broadly balanced between arms.

### Pharmacokinetics and Immunogenicity

**Figure 1:**
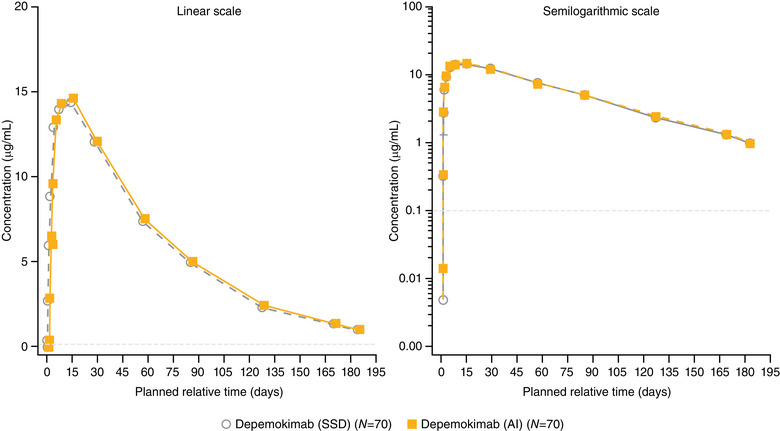
Mean plasma depemokimab concentration–time plots (linear and semilogarithmic) by treatment arm (PK population). LLOQ = 0.1 µg/mL. All BLQ values have been entered as 0 and included in the analysis as such. AI, autoinjector; BLQ, below limit of quantification; LLOQ, lower limit of quantification; PK, pharmacokinetic; SSD, safety syringe device.

Mean plasma concentration–time profiles of depemokimab were similar in both treatment arms in terms of peak concentrations and rate of elimination (Figure 1). The adjusted geometric means (AI:SSD) for C_max_ and AUC_0‐inf_ were 1.03 and 1.03, respectively; all 90% CIs were within the bioequivalence bounds of 0.80‐1.25. The %AUC_ex_ was small and below 20%, with a mean of 5.78 in the SSD arm and 5.68 in the AI arm. Mean arithmetic and geometric means for PK endpoints are shown in Table 1; PK parameters were comparable across treatment arms.

**Table 1:** Mean Arithmetic and Geometric Ratios for PK Endpoints (PK Population)

	Depemokimab SSD (N = 70)	Depemokimab AI (N = 70)
AUC_0‐inf_ (day•µg/mL)		
N	70	69
Arithmetic mean (SD)	1095 (259)	1118 (288)
Geometric mean (%CVb)	1061 (26.9)	1079 (28.4)
Adjusted geometric mean^a^ (SE)	1056 (0.03)	1083 (0.03)^b^
AI/SSD (90% CI)	1.03 (0.96‐1.10)	
C_max_ (µg/mL)		
N	70	70
Arithmetic mean (SD)	15.1 (3.5)	15.4 (3.8)
Geometric mean (%CVb)	14.7 (24.2)	15.0 (23.3)
Adjusted geometric mean (SE)	14.6 (0.0)	15.0 (0.0)^c^
AI/SSD (90% CI)	1.0 (1.0‐1.1)	
AUC_0‐t_ (day•µg/mL)		
N	70	70
Arithmetic mean (SD)	1030 (240)	1049 (264)
Geometric mean (%CVb)	999 (26.07)	1014 (27.35)
CL/F (L/day)		
N	70	69
Arithmetic mean (SD)	0.10 (0.03)	0.10 (0.03)
Geometric mean (%CVb)	0.09 (26.89)	0.09 (28.39)
t_1/2_ (day)		
N	70	69
Arithmetic mean (SD)	42.5 (8.0)	42.1 (7.9)
Geometric mean (%CVb)	41.5 (24.5)	41.2 (23.5)
t_max_ (day)		
N	70	70
Arithmetic median (min, max)^d^	13.0 (4.0, 28.9)	12.4 (3.9, 28.0)
Geometric mean (%CVb)	NA	NA
Vz/F (L)		
N	70	69
Arithmetic mean (SD)	5.80 (1.42)	5.70 (1.44)
Geometric mean (%CVb)	5.64 (25.02)	5.51 (27.23)

AI, autoinjector; AUC_0‐inf_, area under the concentration–time curve from time zero extrapolated to infinity; AUC_0‐t_, area under the concentration–time curve from zero to time of the last quantifiable concentration; CI, confidence interval; CL/F, apparent clearance following subcutaneous dosing; C_max_, maximum observed concentration; CVb, between subject variability; PK, pharmacokinetic; SD, standard deviation; SE, standard error; SSD, safety syringe device; t_1/2_, terminal phase half‐life; t_max_, time at which maximum observed concentration was observed; Vz/F; apparent volume of distribution following subcutaneous dosing.

^a^ The estimates of the geometric means were adjusted for injection site (abdomen, arm, thigh) and baseline weight (log scale).

^b^ Data available for 68 participants.

^c^ Data available for 67 participants.

^d^ Minimum/maximum data presented to 1 decimal place in line with the precision of recordings.

Overall, more than 99% of participants were negative for ADA. At baseline, 1 participant in the SSD arm tested positive for ADA and NAb and continued to test positive for both through Week 26; therefore, this participant was considered to be negative for depemokimab‐related ADAs. One participant in each of the SSD and AI arms tested positive for ADA only at Week 26; the participant in the AI arm also tested positive for NAb, while the participant in the SSD arm tested negative.

### Safety

The most frequently reported AEs (3% or more of participants) are shown in Table 2; headache was the most common AE across both arms. No AEs led to study withdrawal. Treatment‐related AEs were experienced by 19% and 20% of participants in the SSD and AI arms, respectively. Overall, 9 (13%) participants in the SSD arm and 8 (11%) participants in the AI arm reported AESIs. All AESIs were categorized as local injection site reactions, primarily injection site swelling, erythema, or bruising. One participant in the AI arm experienced a local injection site reaction that progressed to a mild systemic Type I hypersensitivity reaction reported as injection site urticaria, which resolved without further intervention. No Type III hypersensitivity reactions were reported.

**Table 2:** Summary of the Most Frequently Reported (≥3%) AEs (Safety Population)

	Depemokimab SSD (N = 70), n (%)	Depemokimab AI (N = 70), n (%)
Any AE	33 (47)	38 (54)
Nervous system disorders		
Headache	7 (10)	6 (9)
General disorders and administration site conditions		
Injection site bruising	3 (4)	1 (1)
Injection site swelling	1 (1)	4 (6)
Injection site erythema	2 (3)	3 (4)
Gastrointestinal disorders		
Vomiting	3 (4)	1 (1)
Nausea	3 (4)	1 (1)
Abdominal pain	1 (1)	3 (4)
Diarrhea	1 (1)	3 (4)
Renal and urinary disorders		
Pollakiuria (Polyuria)	0	3 (4)
Infections and infestations		
COVID‐19	2 (3)	3 (4)
Viral upper respiratory tract infection	0	3 (4)
Any SAE	0	2 (3)
Infections and infestations	0	1 (1)
Pregnancy, puerperium, and perinatal conditions	0	1 (1)^a^
Any AESI		
General disorders and administration site conditions	9 (13)	8 (11)

Includes AEs reported at any time after dosing.

AE, adverse event; AESI, adverse event of special interest; AI, autoinjector; NAb, neutralizing antibody; SAE, serious adverse event; SSD, safety syringe device.

^a^ Two SAEs of spontaneous abortion were reported in a single participant.

Three SAEs were reported by 2 participants (3%) in the AI arm, but none were considered treatment related by the investigator. Both participants who experienced SAEs were ADA negative. The SAEs included 1 episode of pelvic inflammatory disease in 1 participant and 2 episodes of pregnancy ending in spontaneous abortion in the other participant. For the initial pregnancy episode, the participant had a positive pregnancy test 28 days after dosing as part of routine safety monitoring, and a subsequent pregnancy test was negative 56 days after dosing. The same participant had a second positive pregnancy test 201 days after dosing with a subsequent negative test 233 days after dosing.

Liver chemistry remained within normal parameters for the majority of participants. Three (4%) participants in the SSD arm experienced ALT 3× or more the upper limit of normal (ULN); of these, 1 participant had peak ALT 8× or more the ULN and another had peak ALT 20× or more the ULN, reported as nonserious AEs of “alanine aminotransferase increased” and “aspartate aminotransferase increased.” The other participant had a nonserious AE of “hypertransaminasemia.” The mean time from the first dose to the first ALT 3× or more the ULN was 108 days (range, 57‐183). None of the liver events were considered to be treatment related by the investigator.

No participants developed any new clinically significant electrocardiogram abnormalities. Additionally, no clinically meaningful changes were observed in the QT interval corrected for heart rate using Fridericia's formula, with no differences between devices.

### Other Endpoints

**Figure 2:**
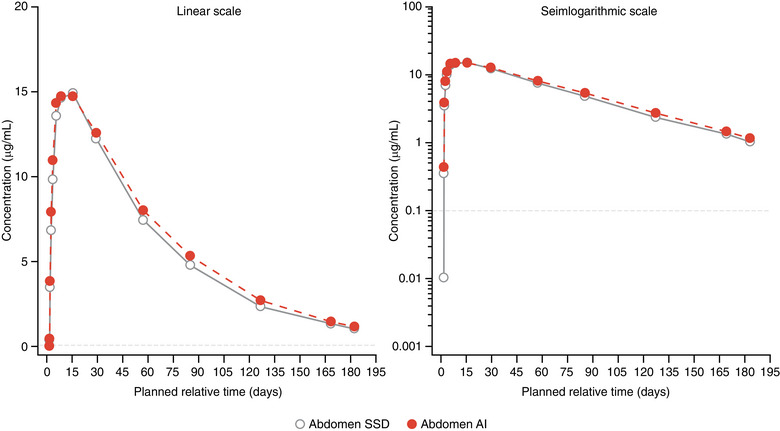
Mean plasma depemokimab concentration–time plots (linear and semilogarithmic) administered in the abdomen by SSD and AI (PK population). LLOQ = 0.1 µg/mL. All BLQ values have been entered as 0 and included in the analysis as such. AI, autoinjector; BLQ, below limit of quantification; LLOQ, lower limit of quantification; PK, pharmacokinetic; SSD, safety syringe device.

**Figure 3:**
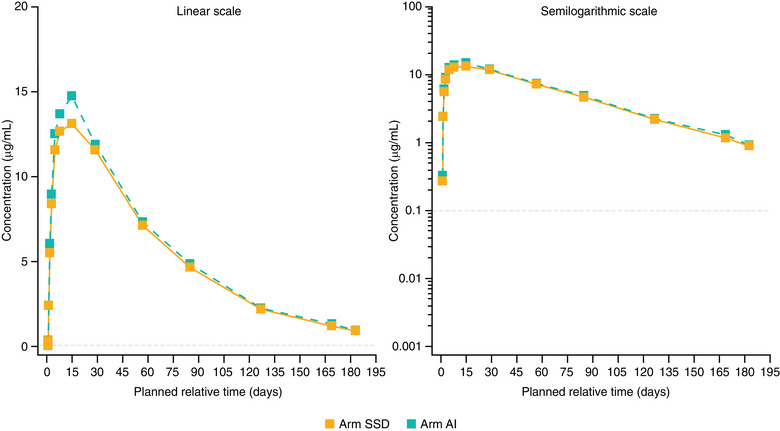
Mean plasma depemokimab concentration‐time plots (linear and semilogarithmic) administered in the arm by SSD and AI (PK population). LLOQ = 0.1 µg/mL. AII BLQ values have been entered as 0 and included in the analysis as such. AI, autoinjector; BLQ, below limit of quantification; LLOQ, lower limit of quantification; PK, pharmacokinetic; SSD, safety syringe device.

**Figure 4:**
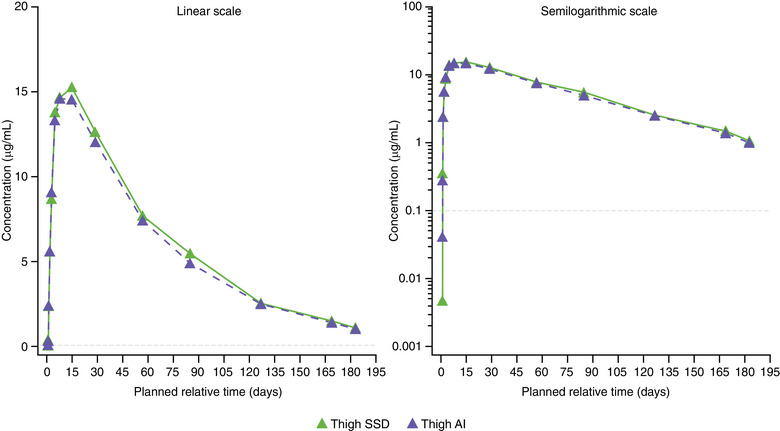
Mean plasma depemokimab concentration–time plots (linear and semilogarithmic) administered in the thigh by SSD and AI (PK population). LLOQ = 0.1 µg/mL. All BLQ values have been entered as 0 and included in the analysis as such. AI, autoinjector; BLQ, below limit of quantification; LLOQ, lower limit of quantification; PK, pharmacokinetic; SSD, safety syringe device.

**Figure 5:**
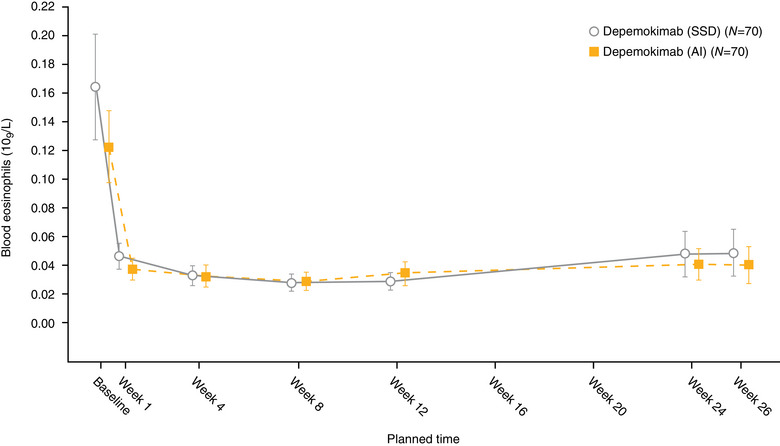
Arithmetic mean blood eosinophils (10^9^/L) by visit (PK population). Error bars indicate 95% confidence intervals. AI, autoinjector; PK, pharmacokinetic; SSD, safety syringe device.

Consistent with the above analysis, mean concentration–time profiles of depemokimab were similar across injection sites, regardless of the device used (Figures 2, 3, 4). C_max_ and AUC_0‐inf_ were similar across the 3 injection sites. Pooled across devices, geometric mean (logSD) C_max_ µg/mL was 15.3 (0.22) for the abdomen, 14.1 (0.25) for the upper arm, and 15.2 (0.23) for the thigh. The geometric mean AUC_0‐inf_ µg•day/mL was 1004 (0.30) for the upper arm, 1119 (0.22) for the abdomen, and 1091 (0.28) for the thigh. Mean blood eosinophil counts at baseline were slightly higher in the SSD than AI arm (arithmetic mean, Figure 5; geometric mean, Figure S1). Both arithmetic and geometric mean ratios to baseline in blood eosinophil counts over time were similar according to the treatment arm although appeared slightly lower in the SSD arm owing to the higher baseline counts. A full dose of depemokimab was successfully administered by site staff to all participants in both arms, indicating no issues with device functionality.

## Discussion

Mean plasma concentration–time profiles were generally similar for both SSD and AI arms following a single dose of depemokimab 100 mg SC, with AI:SSD relative bioavailability close to 1 for both C_max_ and AUC_0‐inf_, and all 90% CIs were contained within the conventional bioequivalence bounds. Consistent with the first‐in‐human study,^3^ plasma concentrations and blood eosinophil reduction over the 26‐week period support a 6‐monthly dosing regimen for both SSD and AI. No variation was observed on the basis of the injection site, regardless of the treatment arm. Both treatment approaches showed a similar safety profile comparable to that of other anti‐IL‐5/IL‐5 receptor antibodies, with few ADAs observed.

SSD and AI devices both offer convenient methods of drug administration, but preferences for each can differ depending on specific patient circumstances. For example, needle fear is prevalent in people with chronic diseases,^12^ and the shrouding of the needle in AIs may reduce the perception of injection pain.^13^ Previous Phase IIIA studies in adolescent/adult patients with asthma have shown that patients/caregivers were able to successfully self‐administer mepolizumab via AI or SSD, with minimal levels of anxiety and a high degree of confidence.^14^, ^15^ When assessing the patient experience with these devices, patients noted the ease of use with both devices, needle retractability for the SSD, and convenience for the AI.^16^ While self‐administration can reduce physician visits, lower patient time burden, and improve quality of life,^17^ it may impact medication adherence and patient experience. Evidence suggests that patients self‐administering at home are less likely to be adherent than those receiving doses in a clinic.^18^ Moreover, patients report that the lack of personal contact with clinical staff is a disadvantage of at‐home administration,^19^ a concern that may increase with extended dosing regimens. Both patients and physicians prefer less frequent dosing schedules for biologics, as they may improve adherence compared with frequent dosing.^2^, ^18^, ^19^ Currently approved biologics for asthma are dosed every 4‐8 weeks.^1^ Given its extended half‐life, depemokimab may offer less frequent dosing. Phase III trials for depemokimab are currently underway for asthma (NCT05243680, NCT04718389), chronic rhinosinusitis with nasal polyps (NCT05274750, NCT05281523), eosinophilic granulomatosis with polyangiitis (NCT05263934), and hypereosinophilic syndrome (NCT05334368). Additionally, the impact of switching to depemokimab from another biologic is currently being assessed in the ongoing Phase III NIMBLE study (evaluating efficacy/safety of switching from mepolizumab/benralizumab to depemokimab), with results expected in late 2026.

Furthermore, flexibility of injection sites may be important for some patients due to factors such as ease of site accessibility during self‐administration or sensitivity of specific sites.^7^, ^11^ PK characteristics of depemokimab did not significantly differ by administration in the upper arm, abdomen, or thigh, regardless of the device used. The availability of multiple injection sites and the option to rotate among them offers patients a further degree of choice in their self‐care.

Strengths of this study include the fact that participants were randomized to devices and sites of administration to limit bias. Also, to reduce potential confounding factors, participants were stratified according to body weight, as this may impact the PK of depemokimab. Limitations include the fact that this was a single‐dose study, which precludes extrapolation for subsequent doses (although it should be noted that AI and SSD should function in the same manner for subsequent doses). Also, this was an open‐label study with no placebo comparator, therefore, no conclusions can be made about the background rates of AEs. Other limitations include the fact that the protocol instructed investigators to examine the injection site at 2 and 48 hours after dosing, which could have resulted in increased reporting of injection site reactions. Additionally, the prespecified BMI limit of 30 kg/m^2^ prevents extrapolation of results for patients with obesity.

## Conclusions

The depemokimab formulation administered via AI has been shown in this study to be bioequivalent to that administered via SSD. The PK characteristics of depemokimab were not affected by the different sites of administration. The incidence of immunogenicity and AEs was low and similar for SSD and AI. Overall, these results support using either SSD or AI devices in depemokimab administration.

## Author Contributions

Stein Schalkwijk contributed to the study concept/design, data acquisition, data analysis, and data interpretation. Chiara Zecchin contributed to the study concept/design and data analysis. Anusmita Sen contributed to the study concept/design, data analysis, and data interpretation. Sei Choi contributed to the study concept/design, data acquisition, and data analysis. Kai Wang contributed to the bioanalytical data acquisition and scientific oversight. Jeff Min contributed to the data acquisition, data analysis, and data interpretation. Brian Spears contributed to the study concept/design and data acquisition. All authors contributed to the drafting of the manuscript, or revised it critically for important intellectual content, gave final approval of the version to be published, and agreed to be accountable for all aspects of the work.

## Conflicts of Interest

S.S., C.Z., A.S., S.C., K.W., and J.M. are employees of GSK and hold financial equities in GSK. B.S. is an employee of PPD, which was contracted by GSK to conduct this study.

## Funding

This study was funded and sponsored by GSK (GSK Study ID: 214099). The sponsor was involved in study design and implementation, as well as data collection, analysis, interpretation, writing the study report, and reviewing this manuscript. All authors had full access to the data upon request and had final responsibility for the decision to submit for publication.

## Supporting information



Supporting Information

## Data Availability

GSK makes available anonymized individual participant data and associated documents from interventional clinical studies that evaluate medicines, upon approval of proposals submitted to https://www.gsk‐studyregister.com/en/.
