# Supplementary material for: Pharmacokinetics of Depemokimab Delivered by Safety Syringe Device or Autoinjector in Healthy Adults: A Phase 1, Single‐Dose Study
Source: Clin Pharmacol Drug Dev. 2025 Jan 28;14(3):190–9. doi: 10.1002/cpdd.1506 (PMC11905874; doi:10.1002/cpdd.1506)
Supplement: Supplementary file 1 — Supporting Information [file CPDD-14-190-s001.pdf]

Pharmacokinetics of Depemokimab Delivered by Safety Syringe Device or Autoinjector in Healthy Adults: A Phase I, Single-Dose Study

Stein Schalkwijk[1], Chiara Zecchin[2], Anusmita Sen[3], Sei Choi[4], Kai Wang[5], Jeff Min[6], Brian Spears[7]

[1]*Clinical Pharmacology Modelling and Simulation, GSK, London, UK*

[2]*Clinical Pharmacology Modelling and Simulation, GSK, Stevenage, Hertfordshire, UK*

[3]*Biostatistics, GSK, Bengaluru, India*

[4]*Global Clinical Development, GSK, Ottawa, Canada*

[5]*Biomarker and Bioanalytical Platform, GSK, Collegeville, PA, USA*

[6]*Clinical Research Respiratory, GSK, Waltham, MA, USA*

[7]*Clinical Research Unit, PPD, Austin, TX, USA*

Corresponding author: Stein Schalkwijk, PharmD, PhD

Email: stein.j.schalkwijk@gsk.com

Postal address: GSK HQ, 79 New Oxford Street, London, WC1A 1DG, UK

Keywords: Biologics; depemokimab; drug metabolism; pharmacokinetics; self-administration

Supplementary Materials

Table S1. Participant characteristics

	Depemokimab SSD N=70	Depemokimab AI N=70	Total N=140
**Female sex, n (%)**	35 (50)	40 (57)	75 (54)
**Age (years) at screening[a]**			
Median (min, max)	37.0 (20.0, 50.0)	35.0 (18.0, 50.0)	36.0 (18.0, 50.0)
Mean (SD)	36.2 (7.8)	34.8 (8.3)	35.5 (8.0)
**Race, n (%)**			
White	45 (64)	43 (61)	88 (63)
Black or African American	15 (21)	22 (31)	37 (26)
Other[b]	10 (14)	5 (7)	15 (11)
**Height (cm), mean (SD)**	169.0 (9.1)	168.5 (9.4)	168.8 (9.2)
**Weight (kg), mean (SD)**	71.9 (11.3)	73.2 (10.9)	72.6 (11.0)
**BMI (kg/m^2)[c], mean (SD)**	25.1 (2.9)	25.7 (2.7)	25.4 (2.8)

AI, autoinjector; BMI, body mass index; SD, standard deviation; SSD, safety syringe device.

[a]Age is imputed when full date of birth is not provided. Percentages are based on the number of participants in each injection site subgroup.
[b]Asian, mixed race, and American Indian or Alaska native.
[c]One participant had a recorded BMI of 30.1 kg/m^2, due to rounding of height and weight measurements, but the actual value was confirmed to be below 30.0 kg/m^2.

Supplementary Figure 1. GMR blood eosinophils (10^9/L) by visit (PK population)

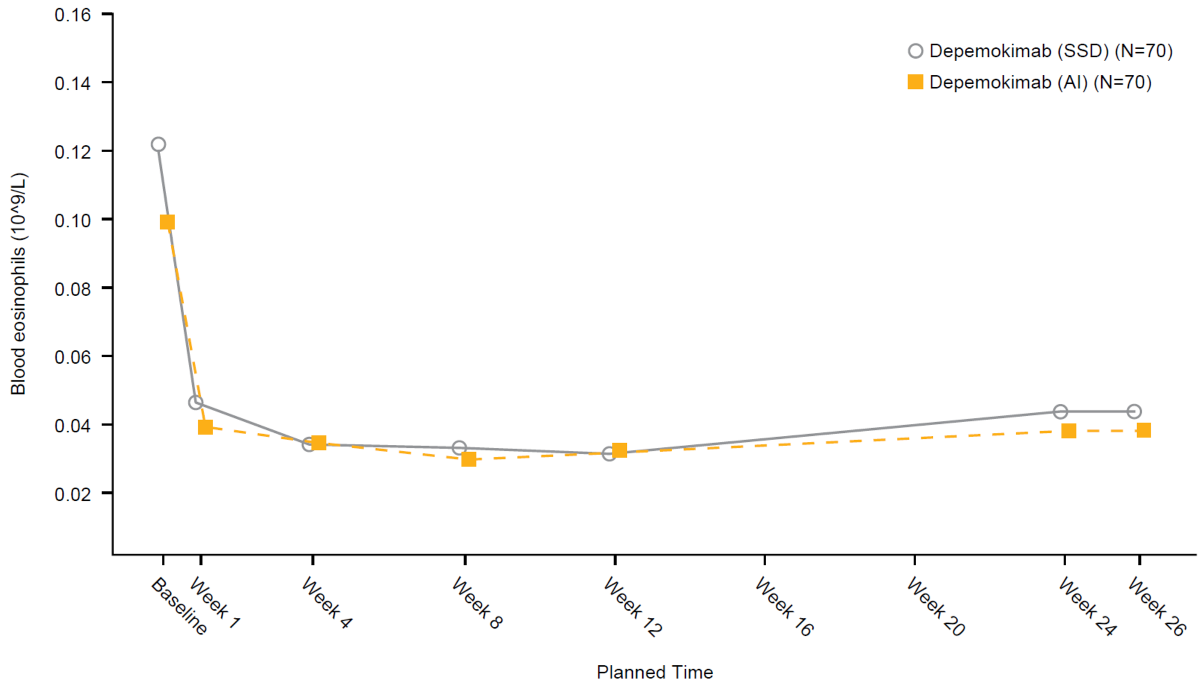

AI, autoinjector; GMR, geometric mean ratio; PK, pharmacokinetic; SSD, safety syringe device.
